# Unlocking the mergers and acquisitions puzzle in the United Arab Emirates: Investigating the impact of corporate leverage on target selection and payment methods

**DOI:** 10.1371/journal.pone.0299717

**Published:** 2024-03-13

**Authors:** Moataz Elmassri, Tariq Z. Elrazaz, Yousry Ahmed

**Affiliations:** 1 Accounting Department, College of Business and Economics, United Arab Emirates University, Al Ain, United Arab Emirates; 2 Accounting Department, Faculty of Commerce, Zagazig University, Zagazig, Egypt; 3 Accounting and Finance Department, Newcastle University, Newcastle upon Tyne, United Kingdom; 4 Business Administration Department, Faculty of Commerce, Zagazig University, Zagazig, Egypt; Huazhong University of Science and Technology College of Management, CHINA

## Abstract

Following a long stream of literature on the drivers of Mergers and Acquisition (M&A) activities, this study examines the effect of corporate leverage on several decisions of M&A deals in the context of the United Arab Emirates (UAE). Using M&A data from the Thomson One database for the period between 2005 and 2022, we find that corporate leverage significantly influences the type of M&A target. This study further adds to the prior literature on the contradictory behaviours of high and low leverage firms by examining whether acquisition decisions differ amongst them in M&A deals in the UAE context. Results indicate that high (low) leverage firms are less (more) likely to acquire private targets and more (less) inclined to acquire a target from a different (same) industry. Furthermore, our results show that the relationship between the method of payment used in M&A deals and corporate leverage is insignificant. We control for endogeneity using Heckman’s two-stage method. In brief, this paper extends the literature with conclusive evidence that considerations of capital structure can significantly anticipate and explain firms’ behaviour toward M&A choices. The implication of findings may include a call to reform some aspects of the Competition Law in the UAE by requiring private firms to enhance their disclosure practices similar to their public counterparts.

## 1. Introduction

To overcome the limitations of organic growth, achieve synergies and maintain competitiveness in a globalized economy, many companies turn to mergers and acquisitions (M&A) as a major corporate finance activity to achieve numerous operational and strategic objectives. M&As also have a substantial impact on capital, labour and product for their potential to create and disseminate value on a large scale into worldwide markets [1–3]. Globally, and over time, M&As have increased both in volume and value. For example, compared to $39 billion in 1987, cross-border M&As in 2016 reached an aggregate $887 billion in value [4]. More recently, the volume of global M&A deals has increased significantly, where in 2021 the value of worldwide M&A deals surpassed USD 5.8 trillion, representing a 64% rise as compared to 2020 and 48% higher than 2018, the previous largest year on record.

Previous studies attempt to answer questions relating to the motivations for the dramatic increase in M&A deals; however, several questions remain unanswered [5–8]. Arguably, the relationship between corporate leverage as a source of business finance and future M&A choices is apparent, surprisingly; little research has examined this link [9, 10]. Prior literature has identified leverage as a key determinant of corporate investment [6], consistent with the notion that highly leveraged firms are more limited in their capacity to raise additional funds [11–14]. To this end, this study investigates whether leveraged firms exhibit symmetric or different behaviours when involved in M&A transactions according to such degree of leverage (i.e., high vs. low). Specifically, we examine the influence of corporate leverage on M&A target selection, including private versus public targets, industrial diversifying versus within-industry acquisitions, as well as the payment methods employed in M&A transactions within the UAE. By examining these factors, we introduce valuable insights into the relationship between corporate leverage and M&A decisions, shedding light on how leverage levels may play a role in a firm’s M&A strategy in the UAE context. Against this backdrop, we address the following research questions:

1. Is corporate leverage a determining factor in acquiring a private target?
2. Does corporate leverage influence an acquirer’s choice to undertake a cross-industry acquisition?
3. Does corporate leverage influence the payment method used in an M&A?

The UAE has emerged to become a major financial hub in the Middle East and North Africa (MENA) region. In 2020, M&A deals in the UAE reached USD 20.9 billion in value, an increase of 13% from the previous year. Corporate leverage is also an important aspect of corporate finance in the UAE, with the ratio of total debt to gross domestic product (GDP) in the UAE higher than the average ratio for emerging market economies (Central Bank of the UAE, 2020). This suggests that corporate leverage is a significant factor in the UAE’s economy and that it plays a key role in financing growth and expansion for firms in the region.

Using a sample of 156 completed M&A deals by UAE public firms from 2005 to 2022, we find that corporate leverage significantly influences the type of M&A target. The findings of the study indicate a negative relationship between corporate leverage and the likelihood of acquiring private targets. Moreover, results also show that corporate leverage has a positive effect on executing industrially diversified acquisitions. However, the effect of corporate leverage on firms’ choice of target type is not symmetric for high- and low-leverage acquirers. In other words, high (low) leverage firms are less (more) likely to acquire private targets. These results are consistent with Capron and Shen’s [15] conjecture that high-leverage firms are more risk-averse and less inclined to invest in private targets, which is as Luypaert [16] posits due to higher information asymmetry about their fair value. Additionally, high (low) leverage firms are more (less) directed towards acquiring a target from a different (same) industry, stemming from the notion brought forward in co-insurance theory that high-leverage firms may consider industrially diversified acquisitions as a means of facilitating their access to funding sources, hence reducing their risk of default [17–19]. Finally, results of this study do not find a significant relationship between corporate leverage and the method of payment used in M&As.

As far as the literature on M&As is concerned, research on corporate leverage within the context of M&As in the UAE is under-presented. We aim to extend the prior literature by filling this gap by examining the impact of corporate leverage on target selection and payment methods in UAE M&A deals. By doing so, we provide important insights into the factors that drive M&A decisions and the role of corporate leverage in these decisions. Moreover, this study also presents invaluable suggestions that acquirers should consider the level of leverage when they choose between private and public targets in an M&A. In particular, high-leverage firms tend to avoid private acquisitions, providing support for information economics theory, which suggests that high-leverage firms may be averse to information asymmetry and prefer public targets with more publicly available information [15].

Moreover, this study offers new evidence that a co-insurance effect linked to acquiring targets from a different industry may be one of the primary reasons for high-leverage firms’ involvement in these deals. Furthermore, this study presents interesting evidence that the level of corporate leverage is a key determining factor in an acquiring firm’s decision in terms of which targets are of potential interest to takeover. We provide evidence that high-leverage acquirers prefer to acquire publicly listed targets from different sectors. On the other hand, low-leverage acquirers choose to acquire private targets from the same sector. Consistent with the literature on M&As, our results indicate that leveraged firms (high vs. low) exhibit differences that may impact various corporate decisions, such as an M&A deal [20, 21]. The present study extends the extant literature by providing further evidence that these differences may even impact firms’ investment choices for M&A deals.

In addition to expanding and contributing to the extant literature on M&As, this study has various implications for the market for corporate control, management practice and academics. First, prior studies in the literature do not segregate the sample of targets according to their public status and attempt to consider all targets belonging to the same industrial sector. In contrast to previous studies [e.g., 6, 7], this study posits that such segregation is both theoretically and empirically warranted since different targets have different characteristics that may be detrimental to the success of an M&A. Empirical findings presented in this article also highlight the importance of corporate leverage level as a key determining factor to be taken into consideration for the market for corporate control since varying levels of leverage among acquirers may impact their investment and acquisition decisions. In other words, this study posits that acquirers characterised by a high level of leverage may have contradictory characteristics that can influence different investment choices as opposed to their low-leverage counterparts.

The empirical findings of this study carry implications for the UAE’s Competition Law, established in 2013 to address anti-competitive practices. Notably, legislators should consider mandating takeover rules that foster a competitive bidding process for private targets, encompassing both acquirers and targets. Such measures have the potential to enhance asset allocation efficiency and promote a more competitive M&A environment, thereby benefiting the broader economy. In this regard, our research extends the corporate finance literature and contributes valuable insights into M&A activity in the UAE, catering to the interests of both practitioners and policymakers. Specifically, our results offer practitioners a heightened understanding of the interplay between corporate leverage and M&A activity, thereby empowering them to make informed decisions when engaging in such transactions. Moreover, policymakers can leverage our research outcomes to formulate more refined and effective regulations and policies governing M&A deals in the UAE context, fostering an environment conducive to sound and strategic corporate decisions. As the body of research progresses, it may be opportune to delve further into the alignment between M&A dynamics and specific aspects of the UAE’s Competition Law, enriching the understanding of the interplay between corporate leverage and M&A decisions in this context.

The remainder of this paper is structured as follows. Section 2 offers a review of the literature. The study methodology, data sources and the empirical models are described in section 3, followed by the empirical results and discussion in section 4. We provide further robustness tests in section 5. Finally, section 6 provides concluding remarks.

## 2. Literature review and hypothesis development

### 2.1 Corporate leverage and firms’ choice between public and private acquisitions

Based on the theoretical perspectives of strategic factor market theory and the information economics theory we can understand acquirers’ decision whether to invest in public or private targets. According to Capron and Shen [15] and Makadok and Barney [22], information economics sees information asymmetry as a source of friction in factor markets, which in turn raises the level of financial risk; on the other hand, strategic factor market theory sees information asymmetry as an opportunity for economic growth [23]. Moreover, it has been documented in the literature that disclosure requirements for private companies, as opposed to their public counterparts, entail a high degree of voluntary reporting requirements, which in turn triggers a heightened degree of uncertainty regarding their true intrinsic value [24]. Moreover, it is well documented in the extant literature that higher levels of debt/leverage result in higher levels of credit risk which in turn deprives companies from raising additional financing from capital markets [13, 25–27]. In his seminal paper, Myers [14] addresses the possible externalities induced by debt on optimal investment strategy to justify the possible relationship between leverage and investments. Indeed, prior studies find evidence indicating that varying levels of leverage may impact a firm’s investment and acquisition decisions [28]. For example, various studies find evidence that leverage is an important factor to consider in issuing equity [29–31], in financing acquisitions [6] and cross-border M&As [32]. Thus, we extend the literature on M&As by questioning whether acquirers characterised by a high level of leverage may have similar or contradictory characteristics that may influence different investment choices, such as acquiring public or private targets, as opposed to their low-leverage counterparts.

As an attempt to explain how and whether corporate leverage affects acquirers’ choices between public and private targets, this study combines perspectives of information economics and strategic factor market theory. According to Humphery et al. [33], Masulis and Simsir [34] and Officer [24], information asymmetry is present between an acquirer and target in all types of M&As. Nevertheless; private targets display a higher degree of information asymmetry as opposed to their public counterparts due to the less opaque nature of disclosed information. This in part is due to the fact that information asymmetry is exacerbated in private targets since they are not required to audit or disclose their financial information [35]. Additionally, managers of private targets have information that investors and acquirers do not have and control the level of disclosure of information they disseminate into the market [36]. As such, there is a certain level of risk that an acquirer may be involved in an adverse target selection and end up overpaying in the acquisition [37, 38]. It follows that, in an attempt to mitigate any negative consequences that may arise from an adverse target selection due to the high degree of risk and level of uncertainty, high-leverage acquirers will prefer to acquire public targets [1].

On the other hand, according to Fama [39], acquirers cannot achieve above-average market returns since public information is readily available to all market participants. In other words, since acquirers have access to comparable information on public targets, investing and competing for the same target seems to be the best decision [23], hence increasing the consideration required, ultimately reducing bidders’ profits to zero [15]. On the other hand, in an acquisition of a private target, information relevant to the deal is exchanged between both parties, which is not the case in contested public acquisitions [40]. This notion discussed above is supported by the findings of Fuller et al. [41], who find that acquirers experience anomalous returns when purchasing public and private targets, respectively. In a similar vein, Faccio et al. [42] report comparable results, where they find that acquirers earn cumulative abnormal returns (CAR) of 1.48% on private acquisitions and a negligible -0.385% on public acquisitions. Against this backdrop, and in line with the assumptions of strategic factor market theory, we posit that low-leveraged firms characterised with low credit risk, will prioritize value-enhancing acquisitions and seek to acquire private targets. Accordingly, the first hypothesis is:

***H1*:** High-leverage firms are less likely to make a private acquisition

### 2.2 Does corporate leverage influence an acquirer’s choice to undertake a cross-industry acquisition?

Another important aspect of an M&A decided by an acquirer is whether the target firm to be acquired operates in the same industry [43, 44]. An acquisition is sought to be industrially diversified in the event of acquiring a target from a different industry. As previously stated, firms with high debt/leverage are deprived to a certain extent of raising additional financing from capital markets. Both Morellec and Zhdanov [21] and Uysal [6] present empirical evidence that highly leveraged acquirers are unwilling to engage in acquisitions due to the fact that such acquisitions would require external financing resulting in higher deal costs.

Pertinent research largely suggests the notion brought forward by co-insurance theory that firms operating in multiple industries exhibit lower earnings fluctuations due to diversified risk which in turn lowers their credit risk and likelihood of default [45, 46]. Almeida and Philippon [18] also show that diversification relieves the counter-cyclical deadweight costs of default risk faced by stand-alone firms. Prior studies in the literature suggest that diversified companies have the capacity to take advantage of economies of scale. A plausible explanation here may be that these companies offer more productive lines of business and more effective operations than stand-alone companies [47–49]. Lewellen [17] and Stein [50] also argue that diversified companies have more debt capacity and debt tax shields in comparison to single-line companies due to the decreased risk associated with diverse companies. Lastly, diversification can result in the creation of internal capital markets, which may lead to an increase in the effectiveness of investments [17, 50]. Therefore, high leveraged acquirers may consider diversified cross-industry acquisitions as a means of reducing their default risk since diversification results in more effective investments which is line with the co-insurance theory [17]. Accordingly the second hypothesis is:

***H2*:** High-leverage firms are more likely to make an industrially diversified acquisition

### 2.3 Corporate leverage and the method of payment in M&As

A key decision in an M&A is the agreement between both parties as to the method of consideration sought in the acquisition. The consideration paid may range from a full cash payment, a pure share payment, to asset/debt swaps [42, 51–53]. According to the pecking-order theory, companies that face higher challenges in attracting outside capital (financially constrained firms) rely on forms of financing that are expected to be less impacted by market inefficiencies in order to reduce their levels of underinvestment. Prior literature finds evidence suggesting that issuing debt is preferred over issuing stocks in financing acquisitions [54]. Initially, due to M&A size and value, these deals are always financed from external sources of finance [55]. According to Almeida et al. [56], Faulkender and Wang [57], and Denis and Sibilkov [58], financially constrained firms have more cash reserves than firms with fewer frictions which they can use for financing acquisitions. In mergers and acquisitions, factors such as the additional cost of debt and the preference for holding cash influence the method of payment chosen to finance the deal [59]. Alshwer et al. [59] find that acquirers who are financially constrained are, on average, less likely to select cash as the method of payment and more likely to use stock as the method of payment than unconstrained acquirers. Similarly, Martynova and Renneboog [12] provide further evidence that a bidder’s financing decision in an M&A can be explained under the premise of pecking order preferences. An additional explanation is presented by Bolton and Freixas’s [60]. Riskier businesses tend to favour bank loans over financing via equity when the capital market is in equilibrium. This is due to the fact that banks are skilled in assisting businesses that are experiencing financial difficulties. That is to say, businesses that face a significant possibility of going bankrupt are more prone to cultivate close lending connections with financial institutions. As such, they have access to the most cost-effective source of financing that is also flexible. The cost of intermediation that is linked with bank loans is avoided by safer companies by issuing equity and bonds instead of taking out bank loans [12]. Owing to the discussions brought forward and in line with prior literature, this study posits that high-leverage firms will be inclined to finance an acquisition using equity via issuing stock as opposed to their low-leverage counterparts who may resort to cash as a method of payment. This is in part due to the fact that low-leverage firms face less financial constraints and a lower degree of risk, which in turn grants them access to cheaper forms of financing [61]. Indeed, prior studies in the literature provide evidence that financially constrained firms (high-leverage) rely on stock as a method of payment in M&As [6, 13]. Against this backdrop, we extend the corporate leverage and M&As previous studies by examining whether financing frictions affect the consideration sought in the acquisition. Accordingly the third hypothesis is:

***H3*:** High-leverage firms are less likely to make cash acquisition deals

## 3. Methodology

### 3.1 Sample

Our paper examines the impact of corporate leverage on target selection in M&As and the method of payment in a sample of 156 M&A deals in the United Arab Emirates (UAE) corporate market. As a starting point, deal information for all completed M&A deals executed by UAE-listed firms during the period 1 January 2005 to 31 December 2022 was extracted from the Thomson One database. Additionally, we obtained financial data of UAE-listed firms from the Eikon database between 2004 and 2022. Our sample selection criteria is consistent with previous research conducted by Barbopoulos et al. [62], Harford et al. [13], Uysal [6], Ahmed and Elshandidy [43], and Ahmed et al. [63]. We only include deals that meet specific requirements, such as completed deals with the acquirer being a UAE publicly listed firm. In line with the literature, we also exclude from our sample acquirers from the financial (SIC 6000–6999) and utilities (SIC 4900–4999) industries due to their unique characteristics and regulatory requirements [64–67]. Additionally, the sample includes both public and private targets. Moreover, the acquisition ends with a wholly owned or holding a majority interest in the target company [68, 69]. Moreover, only deals where the medium of exchange was pure cash, pure stock-for-stock, or a combination thereof are included [69]. Finally, we exclude special forms of deals such as restructuring, and three-way M&As of remaining interest which do not specifically satisfy the definition of a merger or acquisition. Ultimately, our final sample is comprised of 156 successful acquisition deals.

### 3.2 Empirical models

To examine the first research question examining the effect of corporate leverage on the probability of pursuing private acquisitions, we run the following logit model (Eq 1)

$$P(private\;acquisition=1)=\Phi (\beta_{0}+\beta_{1}Leverage_{i,t‐1}+\sum \beta_{i}Controls_{i,t‐1})$$ (1)

*i* denotes the firm, *j* denotes the acquisition deal and *t* denotes the year. A logit model is employed in Eq 1 since our dependent variable (private acquisitions) is a dummy variable that takes the value one if a firm acquires a private target and zero otherwise. Following prior literature, Leverage, which is our main explanatory variable of interest is calculated as the ratio between total debt and total assets [70–73]. We also use an alternative proxy for leverage defined as total debt to total equity; nevertheless, our results are robust to this alternative measure of leverage.

To investigate the second research question examining the effect of corporate leverage on the probability of an acquirer being involved in an industrial diversifying acquisition, we run the following logit model (Eq 2)

$$P(Industrial\;diversifying\;acquisition=1)=\Phi (\beta_{0}+\beta_{1}Leverage_{i,t‐1}+\sum \beta_{i}Controls_{i,t‐1})$$ (2)

A logit model is applied since our main dependent variable (industrially diversified acquisition) is a dummy variable that takes a value of 1 if the acquirer and target are from different industries, and 0 otherwise [43, 44, 69].

To test the third research question regarding the method of payment used in M&A deals and corporate leverage impact, the following logit model is used (Eq 3)

$$P(Cash\;acquisition=1)=\Phi (\beta 0+\beta L\;leverage_{i,t‐1}+\sum \beta i\;Controls_{i,t‐1})$$ (3)

The dependent variable takes the value of one for deals where the method of payment used was cash, and zero otherwise. Following prior studies in the literature, in all the above empirical models we control for firm-specific variables found to have a relationship with the method of payment. Firm size is measured as the natural logarithm of sales. Profitability is measured as earnings before interest, tax, depreciation and amortization (EBITDA) divided by the total asset. The market to book (MTB) ratio is measured as market value of equity to book value of equity. The selling expenses/ sales variable is the ratio of selling expenses over sales. For all regressions, industry dummies are added to control for industry-fixed effects and following Petersen [74], year dummies are also added to control for year fixed-effects [69]. Additionally, all regressions are estimated while clustering at the ‘firm’ level to produce Rogers [75] robust standard errors, which are heteroskedasticity and autocorrelation consistent. Consistent with the extant literature, all continuous data variables used in the regression models are winsorized at the 1% and 99% percentiles to reduce the influence of extreme observations and outliers [44, 76–78]. In order to test for multicollinearity amongst independent variables, we calculate a Variance Inflation Factor (VIF) as well as tolerance values [68]. According to Field [79] and Gujarati [80], VIF values greater than 10 or tolerance values lower than 0.10 signal the existence of harmful multicollinearity in the model. Our testing results indicate that across all our regression models, VIF values are lower than 10 and tolerance values are greater than 0.10, thus we can conclude that none of our models suffers from a problem of multicollinearity.

To provide robustness to our results, we attempt to run our models for a second time while proxying for leverage using an indicator variable that takes the value one if the leverage ratio of the acquirer is higher than the median leverage for the overall sample, and zero otherwise [81].

## 4. Empirical findings and discussion

### 4.1 Descriptive statistics

Table 1 presents descriptive statistics for the variables used in our empirical models for the overall sample. Statistics presented in Table 1 display that over the sample period of 17 years from 2005 to 2022, around 39.7% and 65.2% of UAE public firms decided to be involved in private and industrially diversified acquisitions, respectively. Approximately 10.3% of acquisition deals were financed totally by cash. Table 1 reveals that a total of 10.3% of acquisition deals were financed solely with cash. The mean value of corporate leverage is 0.280 with a standard deviation of 0.208. The deviation around the mean suggests the presence of sub-groups of firms with varying levels of leverage. Moreover, 53.8% of UAE public firms held leverage higher than the sample median per year, as indicated by the high leverage variable in Table 1.

**Table 1:** Descriptive statistics.

	N	Mean	Median	Standard Deviation	Min	Max
Private acquisitions	156	0.397	0.000	0.491	0.000	1.000
Industrial diversifying acquisition	156	0.652	1.000	0.177	0.000	1.000
Cash acquisitions	156	0.103	0.000	0.304	0.000	1.000
Leverage	156	0.280	0.208	0.211	0.000	0.771
High-leverage firms	156	0.538	1.000	0.500	0.000	1.000
Profitability ratio	156	0.075	0.064	0.060	0.108	0.388
Firm size	156	15.001	14.936	1.559	10.258	17.746
MTB	156	0.143	0.113	0.147	0.009	0.919
Selling expenses /sales	156	0.161	0.127	0.153	0.022	0.895

Continuous variables are winsorized at the top and bottom 1 percentile.

### 4.2 Is corporate leverage a determining factor in acquiring a private target?

This section presents empirical results examining how corporate leverage affects a firm’s decision to engage in private versus public acquisitions as presented in Table 2.

**Table 2:** Likelihood of acquiring a private target.

	Private acquisitions	Private acquisitions
	(1)	(2)
Leverage	-0.053***	
	(-4.611)	
High-leverage firms		-1.535***
		(-4.086)
Profitability ratio	-2.910	-0.720
	(-0.952)	(-0.241)
Firm size	-0.135	-0.145
	(-0.871)	(-1.045)
MTB	1.150	1.449
	(0.884)	(1.118)
Selling expenses /sales	-2.227	-1.565
	(-1.630)	(-1.209)
Year FE	Yes	Yes
Industry FE	Yes	Yes
Observations	156	156
Pseudo R-squared	0.156	0.117

(***, **, * represent statistical significance at 1%, 5% and 10% respectively). T-statistics are reported in parenthesis. Standard errors are robust and clustered at the firm level controlling for heteroskedasticity and serial auto-correlation. Industry fixed-effects and year fixed-effects are not reported for sake of brevity. All variables are winsorized at the top and bottom 1 percentile

Column 1 of Table 2 shows that corporate leverage is negatively associated with the probability of acquiring private targets. We further test whether the negative relationship between corporate leverage and the likelihood of private acquisition is identical for both high- and low-leverage firms. Column 2 of Table 2 reports that high-leverage firms have a greater propensity to acquire private targets than public targets. Conversely, low-leverage firms show a preference for private acquisitions. These findings are in line with the information economics view, which considers information asymmetry surrounding private targets as market friction resulting in heightened levels of uncertainty in terms of their value, ultimately rendering them more ambiguous than public targets [15]. Therefore, high-leverage firms that confront higher financial risk are more likely to opt for public acquisitions to avoid any additional risk associated with overpaying for a private target. Additionally, our results align with the strategic factor market theory, which states that low-leverage acquirers characterised by a low credit risk may concentrate on value-enhancing acquisitions of private targets. On the other hand, in an attempt to mitigate any negative consequences that may arise from an adverse target selection due to the high degree of risk and level of uncertainty, high-leverage acquirers will prefer to acquire public targets.

### 4.3 Does corporate leverage influence the likelihood of undertaking an industrially diversified acquisition?

This section presents empirical results examining whether corporate leverage impacts whether an acquiring company seeks to takeover targets belonging to the same industry group, or cross-industry acquisitions are preferred. Results presented in Column 1 of Table 3 show that highly leveraged acquirers prefer diversified cross-industry acquisitions. More specifically, Column 2 of Table 3 shows that high-leverage acquirers invest more in industrially diversified acquisitions rather than acquiring targets belonging to their (acquirers’) same industry.

**Table 3:** Likelihood of making industrial diversifying acquisitions.

	Industrial diversifying acquisition	Industrial diversifying acquisition
	(1)	(2)
Leverage	0.035***	
	(2.593)	
High-leverage firms		1.088**
		(2.166)
Profitability ratio	11.817	-9.779
	(0.410)	(-1.523)
Firm size	0.197	0.142
	(0.680)	(0.497)
MTB	-5.431	1.546
	(-0.498)	(0.528)
Selling expenses /sales	3.181*	2.233
	(1.708)	(1.247)
Year FE	(0.892)	(0.780)
Industry FE		
Observations	156	156
Pseudo R-squared	0.0869	0.0978

(***, **, * represent statistical significance at 1%, 5% and 10% respectively). T-statistics are reported in parenthesis. Standard errors are robust and clustered at the firm level controlling for heteroskedasticity and serial auto-correlation. Industry fixed-effects and year fixed-effects are not reported for sake of brevity. All variables are winsorized at the top and bottom 1 percentile

These findings are consistent with the notion that highly leveraged acquirers may consider diversified cross-industry acquisitions as a means of reducing their default risk which is in line with the co-insurance theory conjuncture of co-insurance theory.

### 4.4 Does corporate leverage influence the payment method used in an M&A?

To address our research question of whether corporate leverage impacts the payment method used in an acquisition, this section provides an empirical analysis of the bidder’s payment choice used in the deal. Table 4 presents results indicating that no statistically significant association is present between corporate leverage and the choice of the payment method used in the acquisition. Column 2 of Table 4 reports the results of our empirical model for high-leveraged acquirers and as shown no significant association has been found between the degree of corporate leverage and cash acquisitions.

**Table 4:** The payment method of an acquisition.

	Cash acquisition	Cash acquisition
	(1)	(2)
Leverage	0.023	
	(0.636)	
High-leverage firms		1.665
		(1.326)
Profitability ratio	-18.329	-13.756
	(-1.558)	(-1.143)
Firm size	0.595**	0.454
	(2.009)	(1.622)
MTB	-1.535	2.998
	(-0.279)	(0.500)
Selling expenses /sales	-4.296	-3.729
	(-0.830)	(-0.597)
Year FE	Yes	Yes
Industry FE	Yes	Yes
Observations	156	156
Pseudo R-squared	0.177	0.210

(***, **, * represent statistical significance at 1%, 5% and 10% respectively). T-statistics are reported in parenthesis. Standard errors are robust and clustered at the firm level controlling for heteroskedasticity and serial auto-correlation. Industry fixed-effects and year fixed-effects are not reported for sake of brevity. All variables are winsorized at the top and bottom 1 percentile

## 5. Robustness checks

### 5.1 Heckman two-step procedure to address self-selection bias

A potential bias from using the OLS method is that a firm’s decision to hold either a high or low leverage level is unlikely to be exogenous. Thus, to account for self-selection bias, we utilize Heckman’s two-step estimation method, following the methodology outlined in Borochin et al. [82]. Firstly, we estimate a probit model to determine the likelihood of holding a high leverage level based on several firm-level characteristics, such as firm size, profitability, asset tangibility, non-debt tax shield, and liquidity [8]. Subsequently, we calculate the inverse Mills ratio from the probit model in the first step and use it as an additional variable in our primary analysis. The two-step process allows us to account for potential self-selection bias and generate more reliable estimates of the effect of corporate leverage on the selection of target types, including public versus private acquisitions and industrial diversifying versus within-industry acquisitions. Our results, presented in Table 5 using Heckman’s two-step self-selection correction model, are consistent with those obtained in the main analysis, indicating that our findings are not affected by self-selection bias.

**Table 5:** Heckman model to address self-selection bias.

	Private acquisition	Private acquisition	Industrial diversifying acquisition	Industrial diversifying acquisition
	(1)	(2)	(3)	(4)
Leverage	-0.052***		0.042***	
	(-4.354)		(2.975)	
High-leverage firms		-1.478***		1.028**
		(-3.956)		(2.079)
Profitability ratio	3.042	7.875	47.879***	-31.308***
	(0.426)	(1.136)	(3.865)	(-3.866)
Firm size	-0.805	-1.095	-1.990**	2.548**
	(-1.123)	(-1.592)	(-2.471)	(2.112)
MTB	0.967	1.134	-8.424	2.132
	(0.712)	(0.881)	(-1.026)	(0.665)
Selling expenses /sales	8.468	13.801	37.025**	-32.355**
	(0.769)	(1.284)	(2.421)	(-2.026)
IMR	-8.413	-12.063	-26.783**	29.541**
	(-0.976)	(-1.439)	(-2.331)	(2.011)
Year Fixed Effects	Yes	Yes	Yes	Yes
Industry Fixed Effects	Yes	Yes	Yes	Yes
Observations (N)	156	156	156	156
Pseudo R-squared	0.161	0.127	0.179	0.115

(***, **, * represent statistical significance at 1%, 5% and 10% respectively). T-statistics are reported in parenthesis. Standard errors are robust and clustered at the firm level controlling for heteroskedasticity and serial auto-correlation. Industry fixed-effects and year fixed-effects are not reported for sake of brevity. All variables are winsorized at the top and bottom 1 percentile

### 5.2 Financial crisis effect

In the aftermath of the global financial crisis, which led to a severe contraction of credit markets, firms faced significant difficulties in obtaining external financing, thus hindering their ability to make investments [83]. As a result, firms with high leverage levels may have been particularly affected. Moreover, Grave et al. [84] found a substantial decline in M&A activity during the crisis. Therefore, it is crucial to examine whether the link between corporate leverage and the choice of target type is driven by the financial crisis shock. To address this concern, we include a financial crisis dummy variable in our analysis that equals one for the years 2008 and 2009 and zero otherwise [43]. Our results, presented in Table 6, indicate that the expected relationship between corporate leverage and target types still holds, and our findings are robust to excluding the effects of the financial crisis period.

**Table 6:** Excluding the financial crisis effect.

	Private acquisition	Private acquisition	Industrial diversifying acquisition	Industrial diversifying acquisition
	(1)	(2)	(3)	(4)
Leverage	-0.054***		0.026*	
	(-4.454)		(1.667)	
High-leverage firms		-1.551***		0.930**
		(-4.149)		(2.519)
Profitability ratio	-2.904	-0.726	9.993	-10.983**
	(-0.961)	(-0.246)	(0.401)	(-2.025)
Firm size	-0.164	-0.150	0.107	0.064
	(-1.058)	(-1.076)	(0.287)	(0.162)
MTB	1.186	1.445	-0.892	1.508
	(0.932)	(1.109)	(-0.225)	(0.679)
Selling expenses /sales	-2.633*	-1.798	1.713	1.089
	(-1.900)	(-1.389)	(0.796)	(0.409)
Financial crisis	-0.093	-0.111	-0.276	-0.343
	(-0.177)	(-0.214)	(-0.250)	(-0.277)
Industry FE	Yes	Yes	Yes	Yes
Observations	156	156	156	156
Pseudo R-squared	0.150	0.115	0.0501	0.0986

(***, **, * represent statistical significance at 1%, 5% and 10% respectively). T-statistics are reported in parenthesis. Standard errors are robust and clustered at the firm level controlling for heteroskedasticity and serial auto-correlation. Industry fixed-effects and year fixed-effects are not reported for sake of brevity. All variables are winsorized at the top and bottom 1 percentile

## 6. Conclusions

This paper investigates the relationship between corporate leverage and the M&A puzzle in the UAE. The results of our study have provided important insights into the factors that drive M&A decisions and the role of corporate leverage in these decisions. Our findings contribute to M&A literature in several ways. Specifically, we find that corporate leverage is a key determinant of target selection in M&A deals. High-leverage firms tend to avoid private acquisitions and are more likely to acquire public or different industry targets, while low-leverage firms tend to undertake private or same industry targets. Our findings extend prior literature by demonstrating that the selection of target types is significantly influenced by a firm’s leverage level. In summary, our paper highlights the significance of considering heterogeneity in firms’ leverage levels when analysing M&A activity.

Furthermore, our study has important implications for academics, practitioners, and policymakers. Our findings emphasize the importance of distinguishing between public and private acquisitions, as well as industrial diversifying and focused acquisitions when studying M&A activity. We also highlight the need to consider the heterogeneity of firms’ leverage when analysing M&A decisions. By providing valuable insights into these issues, our research can aid practitioners in making informed decisions regarding M&A activity, while policymakers can use our findings to develop better regulations and policies surrounding M&A deals in the UAE.

Despite the contributions and insights provided by this study, it is important to acknowledge certain limitations that may warrant further consideration in future research. Firstly, the data used in this research exclusively covers M&A deals involving UAE public firms within the specified timeframe. This may limit the generalizability of the findings to private acquirers or other contexts with distinct institutional and cultural characteristics. Thus, future studies could explore cross-country analyses to gain a more comprehensive understanding of how corporate leverage impacts M&A decisions in diverse global settings. Second, our study examines the impact of corporate leverage on M&A decisions without considering potential variations in economic cycles or market conditions. Future research may explore the interplay between corporate leverage and M&A choices during different economic phases to elucidate how firms’ strategies evolve in response to changing economic landscapes. Finally, future research could also extend this study by examining other factors that may influence the choice of target selection and payment methods in M&A deals, such as corporate governance or market conditions.

## Supporting information

S1 Appendix Definitions of variables. (DOCX)

S1 Data (DTA)
