# Supplementary material for: Unlocking the mergers and acquisitions puzzle in the United Arab Emirates: Investigating the impact of corporate leverage on target selection and payment methods
Source: PLoS One. 2024 Mar 13;19(3):e0299717. doi: 10.1371/journal.pone.0299717 (PMC10936798; doi:10.1371/journal.pone.0299717)
Supplement: S1 Appendix — (DOCX) [file pone.0299717.s001.docx]

**Appendices**

**Appendix A: Definitions of variables**

| **Variable** | **Definition** | **References** |
| --- | --- | --- |
| Cash acquisitions | Takes the value of one for deals where the method of payment used was cash, and zero otherwise. | Ahmed et al. (2023) & Uysal (2011) |
| Firm size | The natural logarithm of sales | Gao & Bao (2022) |
| High-leverage firms | Takes the value one if the leverage ratio of the acquirer is higher than the median leverage for the overall sample, and zero otherwise | Gharsalli (2019) |
| Industrial diversifying acquisition | Takes a value of one if the acquirer and target are from different industries, and zero otherwise | Ahmed & Elshandidy (2016) |
| Leverage | Total debt to total assets | Elsayed et al. (2023) & Louis & Sun (2016) |
| MTB | Market value of equity to book value of equity | Erel et al. (2015) |
| Private acquisitions | Takes the value one if a firm acquires a private target and zero otherwise | Ahmed & Elshandidy (2021) & Capron & Shen (2007) |
| Profitability ratio | Earnings before interest, tax, depreciation and amortization (EBITDA) divided by the total asset. | Harford et al. (2009) |
| Selling expenses /sales | The ratio of selling expenses over sales | Ahmed & Elshandidy (2021) |
